# Correlation of Dielectric Properties and Vibrational Spectra of Composite PVDF/Salt Fibers

**DOI:** 10.3390/polym16172412

**Published:** 2024-08-26

**Authors:** Rashid Dallaev, Ranjini Sarkar, Daud Selimov, Nikola Papež, Pavla Kočková, Richard Schubert, Klara Častková, Farid Orudzhev, Shikhgasan Ramazanov, Vladimír Holcman

**Affiliations:** 1 Department of Physics, Faculty of Electrical Engineering and Communication, Brno University of Technology, Technická 2848/8, 616 00 Brno, Czech Republic; nikola.papez@vut.cz (N.P.); xsneubauerovap@vut.cz (P.K.); xschub01@vut.cz (R.S.); holcman@vut.cz (V.H.); 2 Department of Metallurgical and Materials Engineering, Indian Institute of Technology Kharagpur, Kharagpur 721302, West Bengal, India; ranjinisarkar.kgp.1991@gmail.com; 3 Department of Inorganic Chemistry and Chemical Ecology, Dagestan State University, St. M. Gadjieva 43-a, 367015 Makhachkala, Russia; farid-stkha@mail.ru (F.O.); daud-selimov@live.com (D.S.); 4 Central European Institute of Technology BUT, Purkyňova 123, 612 00 Brno, Czech Republic; klara.castkova@ceitec.vutbr.cz; 5 Amirkhanov Institute of Physics, Dagestan Federal Research Center, Russian Academy of Sciences, 367003 Makhachkala, Russia; ramazanv@mail.ru

**Keywords:** PVDF, nitrate salt, XRD, Raman spectroscopy, FTIR

## Abstract

Nitride salts were added to polyvinylidene fluoride fibers and then the fiber mats were prepared by electrospinning. An experimental investigation of the structure was provided by Raman, FTIR, SEM, and XRD. The phase ratio of the polymer was studied both theoretically and experimentally in connection with the addition of the hydrates Mg(NO_3_)_2_, Ca(NO_3_)_2_, and Zn(NO_3_)_2_ salts. The comparison of simulated and experimental data for vibrational spectroscopies is discussed. We provide a comparison of triboelectric, dielectric, and compositional characterization of PVDF fibers doped with three types of nitride hydrates. Doping of PVDF fibers with magnesium nitrate hexahydrate leads to significant improvement of the triboelectric performance.

## 1. Introduction

Doping of piezopolymers with functional additives is a way to create smart and responsive materials [1]. PVDF fibers loaded with nitrate salts have several potential applications, particularly in the field of energetic materials, sensors, and biomedical devices. The combination of PVDF nanofibers and nitrate salts can offer unique properties and functionalities, making them suitable for various practical uses. The combination of PVDF’s high mechanical strength and piezoelectricity with the energy release properties of nitrate salts can lead to improved performance of energetic materials or propellants in rocket propulsion systems and other propulsion devices [2,3]. By integrating nitrate salts into PVDF, it is possible to prepare the composites for sensors that are capable of detecting pressure, strain, and mechanical vibrations and find practical applications in structural health monitoring, wearable devices, and smart textiles [4]. When PVDF nanofibers are combined with nitrate salts, they exhibit potential in drug delivery systems and tissue engineering scaffolds [5]. Furthermore, their piezoelectric properties enable applications in nerve regeneration and as sensors for biological signals [6]. The amalgamation of PVDF nanofibers and nitrate salts opens avenues in pyrotechnics and fireworks, creating unique visual effects through the energetic release of the salts upon ignition. PVDF nanofibers loaded with nitrate salts find utility in environmental monitoring devices, capable of detecting hazardous substances. Additionally, they can be integrated into safety systems for identifying gas leaks or explosive materials [7,8].

Dielectric properties refer to a material’s ability to store and dissipate electric energy. Key parameters include the dielectric constant (permittivity) and dielectric loss. These properties are influenced by molecular structure, polarizability, and the interaction of molecules with an electric field. Vibrational spectroscopies involve passing radiation through a sample and recording the absorbed wavelengths. The resulting spectrum provides information about the molecular vibrations, which are related to the functional groups and molecular structure of the material.

Dielectric properties are related to molecular motions such as dipole relaxation and vibrations. Vibrational spectroscopies can help identify these motions and their contributions to energy dissipation. Triboelectric charging, also known as contact electrification, is a widely recognized and frequently observed phenomenon. It occurs when material fibers repeatedly come into contact and then separate, causing charge transfer between surfaces. This transfer is influenced by both kinetic and equilibrium effects. PVDF in particular tends to gain a negative charge when it comes into contact with other materials.

In this paper, we suggest a theoretical and experimental approach for the explanation of the composite formation through modeling and experimental investigation of vibrational spectra. These studies contribute to the evaluation of the dielectric properties of the polymeric fibers which defines their application potential. We present XPS and SEM analyses because the surface condition is crucial for triboelectric response. Electrical characterization is necessary for electrospun fiber mats to facilitate their development and commercialization.

Specific types of nitrate hydrates were chosen, where the compatibility with PVDF and mechanism of interaction was previously studied [9]. Earlier we reported the functional properties of these composites [10]. In this work, we are focused on understanding PVDF phase conformations in dependence on *salt cations*. The novelty of the research consists of a comparison of theoretical and experimental approaches for the phase’s investigation for the explanation of the dielectric properties of the composites.

## 2. Materials and Methods

### 2.1. Sample Preparation

The polyvinylidene fluoride (PVDF) material used in the following measurements was obtained from Sigma Aldrich (St. Louis, MO, USA) and prepared as fibers with a molecular weight of 275,000 g × mol^−1^. The fibers were produced by electrospinning a 15 wt% PVDF solution in a blend of dimethylsulfoxide (Sigma Aldrich, St. Louis, MO, USA) and acetone (Sigma Aldrich, St. Louis, MO, USA) at a volume ratio of 7/3. To this solution, calcium, magnesium, and zinc nitrates (in their hydrated form) from Lach-Ner (Neratovice, Czech Republic) were added at 8 wt% relative to the solid polymer before dissolving the PVDF. The solution was stirred for 24 h at 40 °C.

Electrospinning was conducted using 4-SPIN equipment from Contipro (Dolní Dobrouč, the Czech Republic) at a feeding rate of 20 µL × min^−1^, employing a thin needle with a diameter of 1.067 mm (17 G). The resulting fibers were collected on an aluminum foil-covered rotation collector, spinning at a speed of 2000 rpm for 30 min. The distance between the needle tip and the collector was maintained at 20 cm during the process. The nonwoven fiber mats produced were left to dry overnight at room temperature. The resulting fibers’ diameter ranged from 300 to 700 nm.

### 2.2. Sample Experimental Characterization

Scanning electron microscopy analysis was conducted by electron microscope utilizing a Tescan LYRA3 (Tescan, Brno, Czech Republic). The samples were coated with 10 nm of carbon to avoid charging. The acceleration voltage was 5 kV with a view field of 200 µm and 10 µm. Cross-sectional imaging was performed using a Focused Ion Beam/Scanning Electron Microscope FEI Helios NanoLab 660 (FEI, Brno, Czech Republic). A Ga-focused ion beam was employed with an accelerating voltage of 5 kV and a current of 43 pA for sectioning. The resulting cross-sections were then examined using the SEM capabilities of the same Helios system.

X-ray photoelectron spectroscopy (XPS) was performed to analyze the chemical bonding in the samples using an AXIS Supra instrument (Kratos Analytical Ltd., Manchester, UK). The measurements were taken with an emission current of 15 mA, and the spectra were acquired at a resolution of 20 for wide scans and 80 for element-specific scans. Fourier-transform infrared (FTIR) spectroscopy measurements were conducted to assess the phase composition of the samples using a Bruker instrument (Billerica, MA, USA) in transmission mode, with 512 scans and a resolution of 1 cm⁻¹. X-ray powder diffraction (XRD) analysis was employed to confirm the crystalline structure of the samples, utilizing a Rigaku SmartLab 3 kW system (Rigaku, Tokyo, Japan) configured in the Bragg–Brentano geometry. Diffraction patterns were recorded in the 2θ range of 10° to 25° using Cu Kα radiation. Raman spectroscopy was performed to analyze the structural characteristics of the samples with a WITec alpha300 R system (WITec, Ulm, Germany), operating at an excitation wavelength of 532 nm and a laser power of 10 mW. The Raman signal was averaged over 20 accumulations with an integration time of 10 s per accumulation.

### 2.3. Computational Methods

Density functional theory (DFT) studies were conducted to illustrate the inter-component interactions within PVDF/salt complexes. All the DFT calculations were performed based on the linear combination of atomic orbitals (LCAO) theory using the Gaussian 16 software suite [11]. Hybrid exchange-correlation functional B3LYP (Becke’s nonlocal gradient-corrected three-parameter exchange functional [12] with correlation functional developed by Lee–Yang–Parr [13]) was used with polarized triple-ζ basis set 6-311+G(d,p). Long-range dispersion correction was incorporated by implementing Grimme’s dispersion correction DFT-D3 [14]. Frequency scaling factors of 0.9679 and 0.9877 were applied to obtain the vibrational frequency modes and zero-point energies (ZPE), respectively [15].

To model the PVDF/salt complexes, tetramer chains of three polymorphs of PVDF, i.e., α-, β-, and γ-PVDF were considered. Two configurations (denoted as C1 and C2 elsewhere) of each of the hydrated nitrate salt molecules, i.e., Mg(NO_3_)_2_·6H_2_O, Ca(NO_3_)_2_·4H_2_O, and Zn(NO_3_)_2_·6H_2_O were designed according to their coordination geometries, i.e., the arrangement of the central bivalent cations (Mg^2+^ or Ca^2+^ or Zn^2+^), NO_3_^−^ anions, and the water molecules, based on similar DFT studies on hydrated nitrate salts of different metals reported earlier [16,17]. The formation energies of the two configurations C1 and C2 ($E_{f,C1}$ and $E_{f,C2}$, respectively) were calculated as,

$$E_{f,C1}=E_{C1}-\left(E_{{M({NO}_{3})}_{2}}+{nE}_{H_{2}O}\right)$$

$$E_{f,C2}=E_{C2}-\left(E_{{\left[{M\left(H_{2}O\right)}_{n}\right]}^{2+}}2E_{{\left[{NO}_{3}\right]}^{-}}\right)$$

Here, $E_{C1}$ and $E_{C2}$ denote the ZPE-corrected energies of the two configurations of each salt molecule (C1 and C2), respectively. $E_{{M({NO}_{3})}_{2}}$ and $E_{{\left[{M\left(H_{2}O\right)}_{n}\right]}^{2+}}$ are the ZPE-corrected energies of the central units of C1 and C2, respectively, with M and n denoting the metals (Mg or Ca or Zn) and the number of water molecules, respectively. $E_{H_{2}O}$ and $E_{{\left[{NO}_{3}\right]}^{-}}$ refer to the ZPE-corrected energies of an isolated H_2_O molecule and [NO_3_]^−^ ion, respectively.

PVDF/salt interaction energies ($E_{int}$) were calculated as,

$$E_{int}=E_{PVDF/salt}-\left(E_{PVDF}+E_{salt}\right)$$

Here, $E_{PVDF/salt}$, $E_{PVDF}$, and $E_{salt}$ represent the ZPE-corrected energies of the PVDF/salt complex, isolated PVDF (in α-, β-, or γ-phase) tetramer chain, and isolated salt (C1 or C2) molecules.

### 2.4. Electrical Characterization

To measure the triboelectric properties of materials, a systematic approach involves using a triboelectric generator, variable load resistor, and precise voltage and current measurement tools. The experiment begins by setting up the triboelectric generator, which consists of two different materials that come into contact and then separate, generating an electric charge. The output terminals of the triboelectric generator are connected to a variable load resistor. Initially, the load resistor is set to a very high value to measure the open-circuit voltage, and then to a very low value to measure the short-circuit current. These measurements provide baseline data for the voltage and current capabilities of the triboelectric generator. Subsequently, the load resistance is adjusted incrementally from low to high values. For each resistance value, the voltage across the resistor and the current through it are measured using precision voltmeters and ammeters, with data recorded over multiple cycles to ensure accuracy and repeatability. The measurements were validated and published in previous work dedicated to this method [18]. Dielectric property measurements, crucial for defining sample functionality, were conducted using a Novocontrol Alpha Analyzer device (Novocontrol Technologies, Montabaur, Germany) across a frequency range spanning 1 to 100,000 Hz.

## 3. Results

### 3.1. Sample Morphology

**Figure 1:**
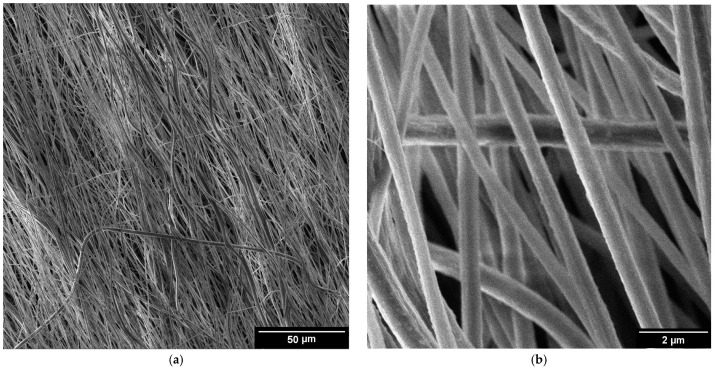

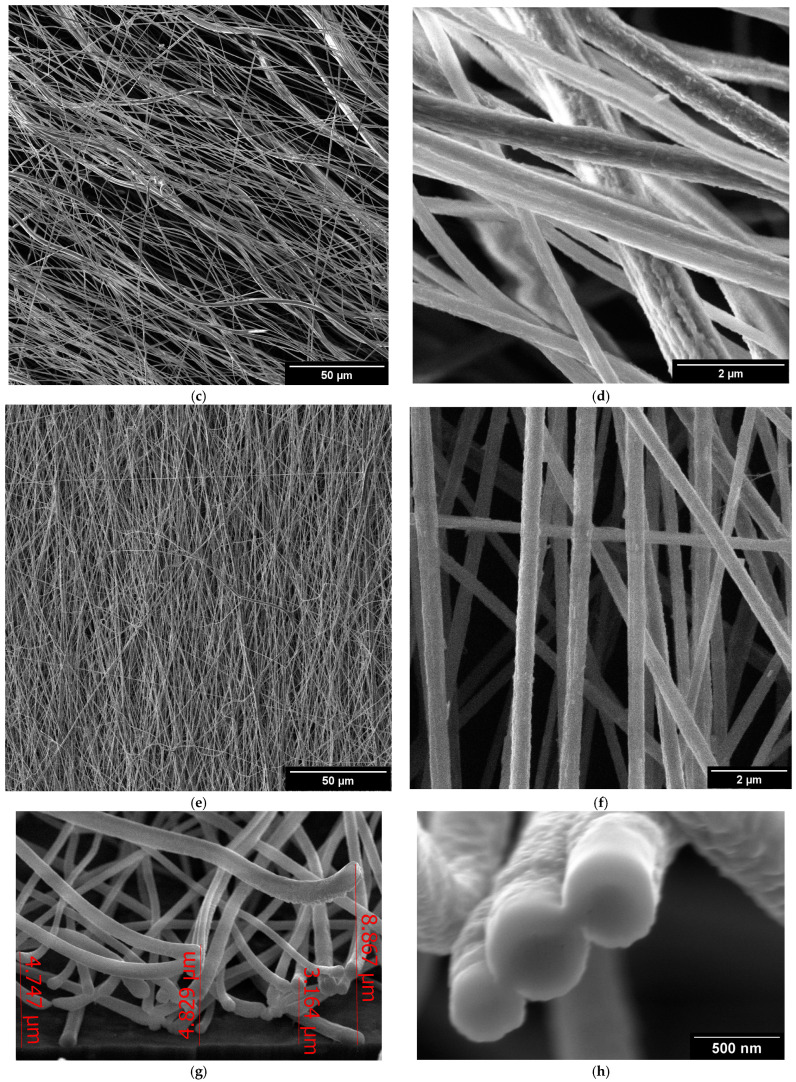
SEM images of salt hydrate-enhanced PVDF fibers: (**a**,**b**) Mg(NO_3_)_2_·6H_2_O, (**c**,**d**) Ca(NO_3_)_2_·4H_2_O, and (**e**,**f**) Zn(NO_3_)_2_·6H_2_O. Cross-section of the Mg(NO_3_)_2_·6H_2_O (**g**) and detailed images of the cut fibers (**h**).

The detailed surface topography of the composite fibers is presented in secondary electron images presented in Figure 1. The fibers were uniform in terms of diameter and surface texture (Figure 1a,c,e). The fibers exhibited an average diameter of 500 nm. The surface of the fibers appeared smooth, with the rare presence of grooves (Figure 1b,d,e). This texture can be attributed to the chosen composition and electrospinning parameters. The thickness of the samples varied between 4 and 8 µm, as illustrated in Figure 1g,h. The images show the cross-section of the Mg(NO_3_)_2_·6H_2_O sample, the general characteristics of the remaining samples exhibit similar features.

### 3.2. Sample Composition

The crystalline structure of the used salt hydrates can be described in terms of the arrangement of its constituent ions and water molecules. The detailed structure is typically determined through X-ray diffraction. The overall structure is stabilized by hydrogen bonds, forming a crystalline lattice.

Magnesium nitrate hexahydrate (Mg(NO_3_)_2_·6H_2_O) and zinc nitrate hexahydrate (Zn(NO_3_)_2_·6H_2_O) crystallize in the monoclinic crystal system. In this structure, the metal ions are surrounded by six water molecules, forming a coordination complex. The nitrate ions are also involved in the crystal lattice, contributing to the overall stability and arrangement of the crystal. Calcium nitrate tetrahydrate (Ca(NO_3_)_2_·4H_2_O) crystallizes in the monoclinic crystal system. The structure typically features calcium ions coordinated by water molecules and nitrate ions, creating a complex network.

**Figure 2:**
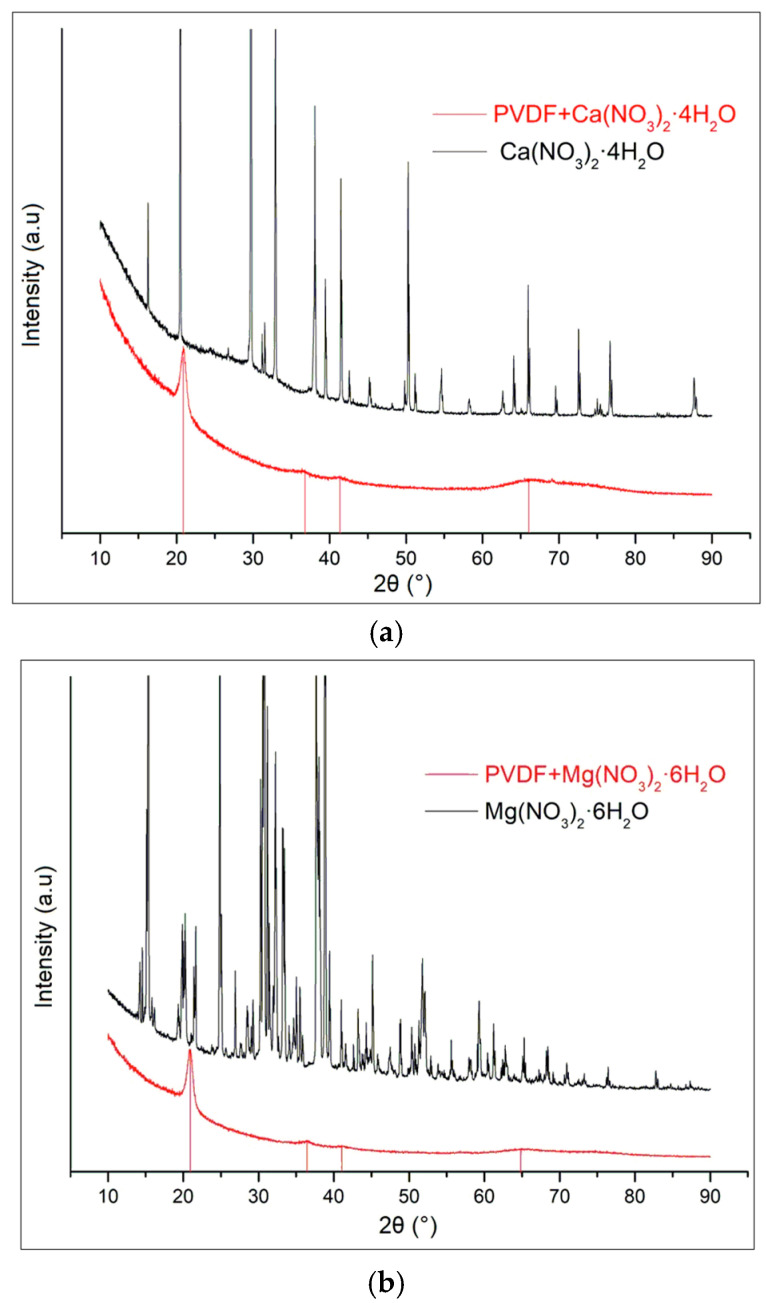

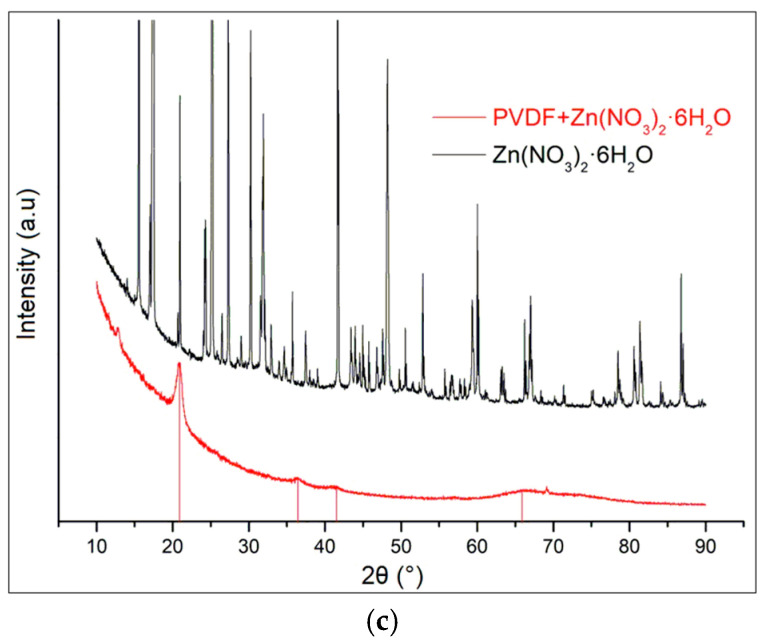
XRD spectra of nitrate hydrates and doped PVDF fibers for (**a**) Ca(NO_3_)_2_·4H_2_O; (**b**) Mg(NO_3_)_2_·6H_2_O; and (**c**) Zn(NO_3_)_2_·6H_2_O.

XRD patterns of the composites sample do not include any diffraction peaks of magnesium nitrate hexahydrate (Figure 2a) [19,20,21], calcium nitrate tetrahydrate (Figure 2b), or zinc nitrate hexahydrate (Figure 2c). The raw data are presented in Supplementary Materials.

**Figure 3:**
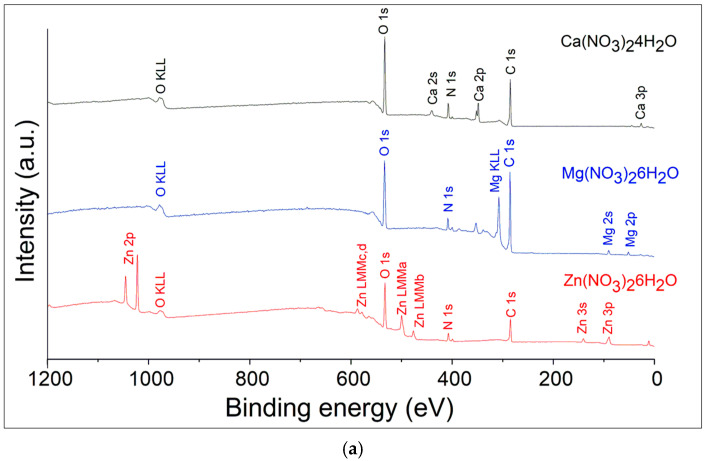

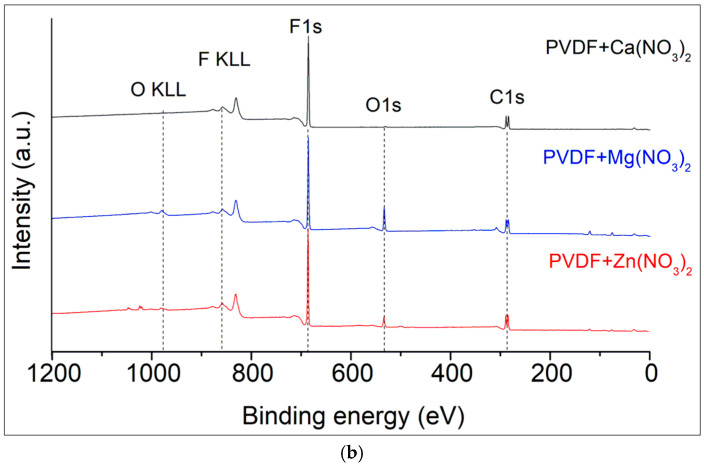
XPS survey spectra of the hydrated salts (**a**) and salt/nanofiber composites (**b**).

The survey XPS spectra cover a wide range of binding energies, from 0 to 1200 eV. The salt spectra are shown in Figure 3a and contain expected ions of metals, nitrogen, oxygen, and contamination of carbon. The survey spectrum displays peaks corresponding to various core levels of the metal elements present in the sample. There are visible peaks of calcium Ca2s, Ca2p, Ca3p for Ca(NO_3_)_2_·4H_2_O; Mg2s, Mg2p, and Mg KLL Auger peak for Mg(NO_3_)_2_·6H_2_O; Zn2p, Zn3s, Zn3p, and four Auger peaks of Zn for Zn(NO_3_)_2_·6H_2_O. The Zn LMM notation specifies the shells involved in the Auger process: the initial hole is in an L shell, and the transitions involve electrons from the M shells. The various possible transitions can lead to different Auger peaks (Figure 3a) depending on the specific subshells involved. These subshells are characterized by different binding energies, leading to distinct Auger electron energies. Here are the potential LMM transitions for zinc: L_3_M_4_M_4_, L_3_M_4_M_5_, L_2_M_4_M_4_, and L_2_M_4_M_5_.

The main peaks include the C 1s and F 1s which correspond to the polymer’s backbone structure, and O 1s peaks which indicate the presence of oxidized elements and oxygen contamination (Figure 3b). All the spectra demonstrate the presence of the F KLL Auger peak at binding energies of 830 eV as a confirmatory signal. Deconvolution techniques to separate overlapping peaks and accurately identify the chemical states of the elements were done by Casa XPS software version 2.3.17PR1.1. Raw data are presented in Supplementary Materials.

**Figure 4:**
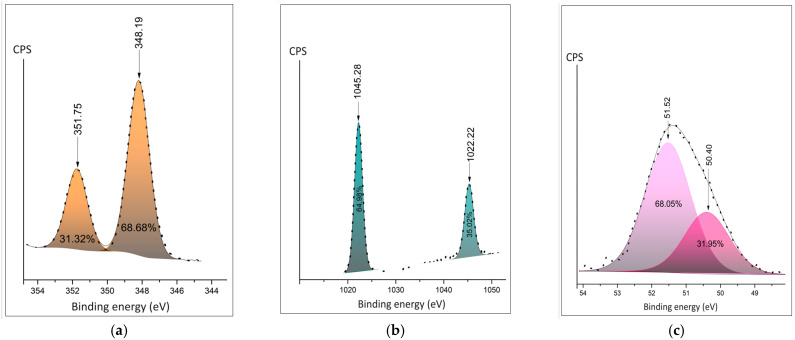

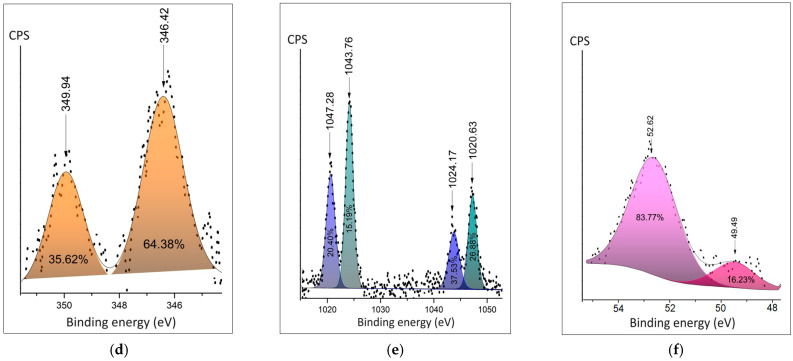
Detailed XPS peaks of the metal cation: (**a**) Ca 2p in the salt hydrate; (**b**) Zn 2p in the salt hydrate; (**c**) Mg2p in the salt hydrate; (**d**) Ca2p peak in the polymer composite; (**e**) Zn 2p peak in the polymer composite; (**f**) Mg2p peak in the polymer composite.

Detailed XPS spectra are presented in Figure 4a–c. The Ca2p duplet peaks move to the lower energies for the Ca^+^ involved in polymer composite (Figure 4a,d). The shape of Zn2p shows the appearance of the second doublet, corresponding to the appearance of an additional oxidation state in polymer composition (Figure 4b,e). Both the position and area of Mg2p ionic bonds show fewer oxygen bonds in the composite samples. The distance between the spin split peaks shown in the figures demonstrates expectable changes in the chemical surroundings of the cations.

**Figure 5:**
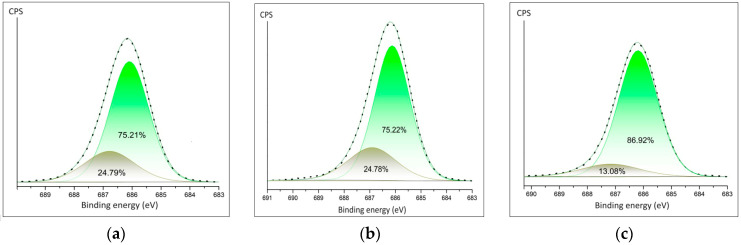
Detailed XPS peaks of the fluorine (**a**) in PVDF fibers doped by Ca(NO_3_)_2_·4H_2_O; (**b**) in PVDF fibers doped by Zn(NO_3_)_2_·6H_2_O; (**c**) in PVDF fibers doped by Mg(NO_3_)_2_·6H_2_O.

The detailed spectra of fluorine are shown in Figure 5a–c. The spectra of PVDF fibers doped Ca(NO_3_)_2_·4H_2_O and Zn(NO_3_)_2_·6H_2_O show similar ratios of the fluorine–carbon and fluorine–metal bonds (Figure 5a,b). The area of the C-F bond is twice as large in PVDF fibers doped Mg(NO_3_)_2_·6H_2_O composite.

**Figure 6:**
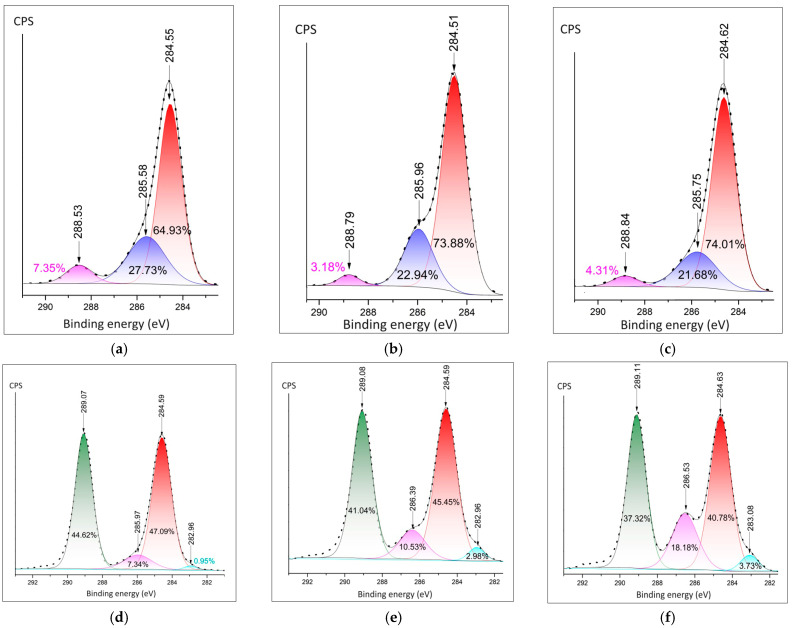
Detailed XPS peaks of the metal cation: (**a**) C1s in the Ca(NO_3_)_2_·4H_2_O; (**b**) C1s in the Zn(NO_3_)_2_·6H_2_O; (**c**) C1s in the Mg(NO_3_)_2_·6H_2_O; (**d**) C1s peak in the PVDF fibers doped Ca(NO_3_)_2_·4H_2_O; (**e**) C1s in PVDF doped by Zn(NO_3_)_2_·6H_2_O; (**f**) C1s in PVDF fibers doped by Mg(NO_3_)_2_·6H_2_O.

Detailed spectra of carbon were measured by the C1s peak. The C-C peak is present at all spectra of hydrates and composites (Figure 6a–f). The significant contribution of the O-C=O bond could be observed at 288.53 eV at Ca(NO_3_)_2_·4H_2_O sample (Figure 6a). The samples of composites have a C-F peak of around 289.1 eV. The lowest rate of C-F in comparison (37.32%) in comparison with the C-C bond is at the sample of PVDF fibers doped by Mg(NO_3_)_2_·6H_2_O. It is in agreement with the F1s spectra (Figure 5c) and confirms the bonding of fluorine with metal.

### 3.3. DFT Analyses

**Figure 7:**
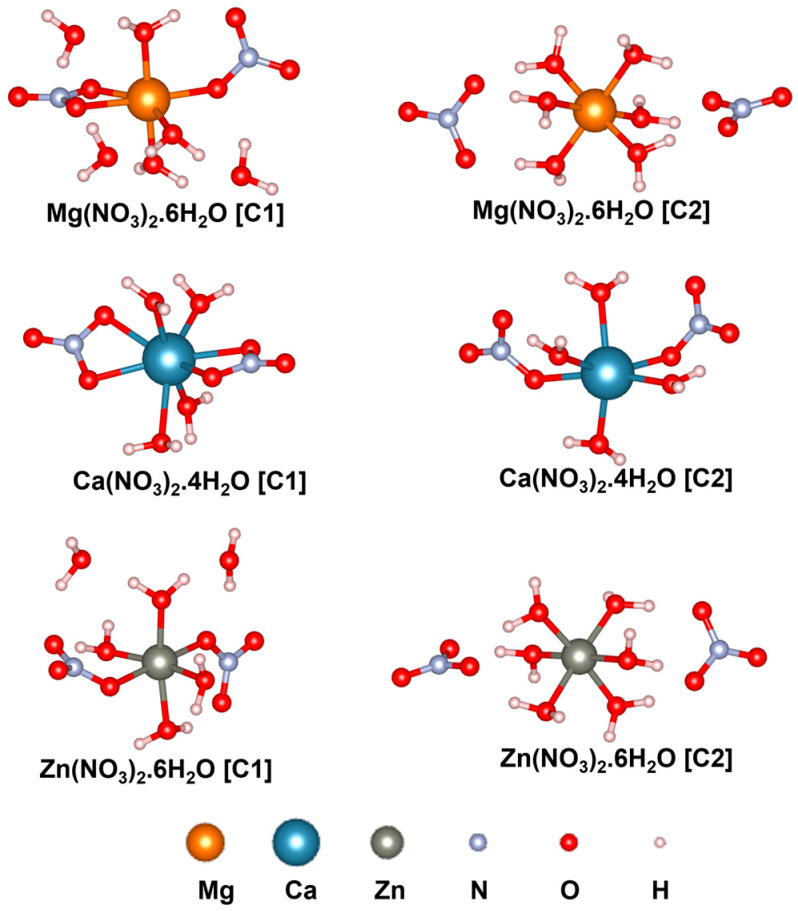
Optimized structures of hydrated salt systems. C1 and C2 represent configurations 1 and 2, respectively.

In this section, the PVDF/salt interaction is elucidated based on first-principles DFT analyses. Optimized structures of the hydrated salt molecules are provided in Figure 7. Within initial configurations (before optimization) of C1 structures, the bivalent metal ions M^2+^ (M: Mg, Ca, Zn) are connected to two [NO_3_]^−^ ions forming [M(NO_3_)_2_] central unit surrounded by water molecules. Conversely, in the initial C2 structures, M^2+^ ions are bonded with water molecules, forming the [M(H_2_O)_n_]^2+^ central unit which is surrounded by two [NO_3_]^−^ ions. Notably, after optimization, both H_2_O and [NO_3_]^−^ moieties can form ionic or coordination bonds with the central metal cation, as depicted in Figure 7. The average Mg-O, Ca-O, and Zn-O bond distances are 2.11, 2.45, and 2.13 Å, respectively, within C1, and 2.09, 2.36, and 2.12 Å, respectively, within C2. The configurational energy differences (${{∆E}_{C}=E}_{C1}-E_{C2}$) and the formation energies ($E_{f,C1}$ and $E_{f,C2}$) of the salt complexes are provided in Table 1. A negative value of ${∆E}_{C}$ indicates better stability of C1 with more negative electronic energy. On the other hand, a positive value of ${∆E}_{C}$ indicates that C2 exhibits more negative electronic energy, i.e., better stability compared to C1. Therefore, as evident from Table 1, Mg(NO_3_)_2_·6H_2_O is more stable as C2 than C1 with ${∆E}_{C}$ of 1.88 kcal/mol. On the contrary, C1 of Ca(NO_3_)_2_·4H_2_O and Zn(NO_3_)_2_·6H_2_O are more stable than the respective C2 structures, with ${∆E}_{C}$ of 1.26 and 1.88 kcal/mol, respectively. Furthermore, $E_{f,C2}$ values are found to be more negative than $E_{f,C1}$ for all the salt complexes. Nevertheless, considering the feasibility of the formation of both salt configurations, as indicated by the negative $E_{f,C1}$ and $E_{f,C2}$ values, we have included both C1 and C2 to model the PVDF/salt complexes.

**Figure 8:**
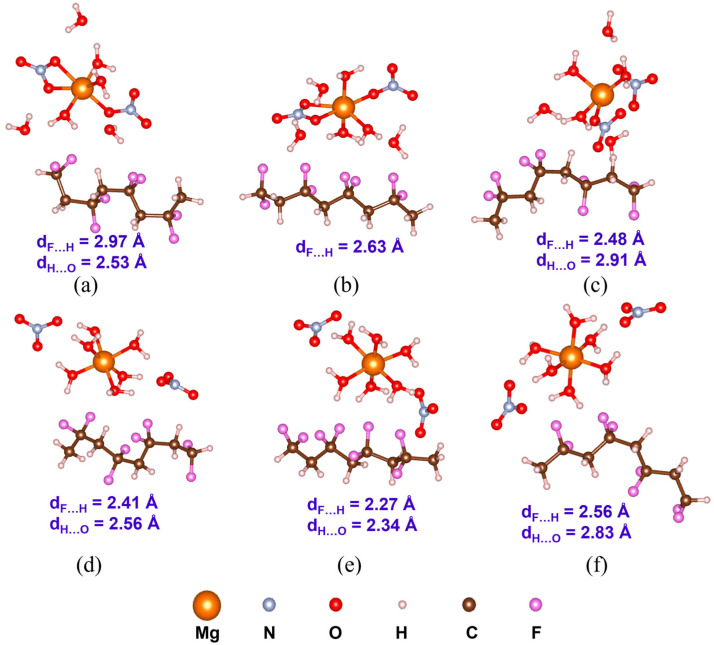
Optimized structures with the average inter-component F…H and H…O distances (d_F…H_ and d_H…O_) of PVDF/Mg(NO_3_)_2_·6H_2_O complexes: (**a**) α-PVDF/Mg-salt (C1), (**b**) β-PVDF/Mg-salt (C1), (**c**) γ-PVDF/Mg-salt (C1), (**d**) α-PVDF/Mg-sal t(C2), (**e**) β-PVDF/Mg-salt (C2), and (**f**) γ-PVDF/Mg-salt (C2).

**Figure 9:**
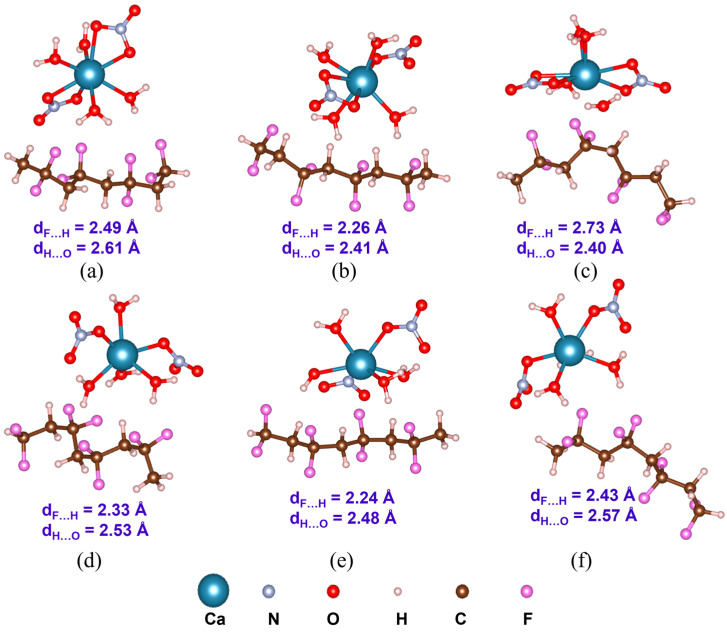
Optimized structures with the average inter-component F…H and H…O distances (d_F…H_ and d_H…O_) of PVDF/Ca(NO_3_)_2_ 4H_2_O complexes: (**a**) α-PVDF/Ca-salt (C1), (**b**) β-PVDF/Ca-salt (C1), (**c**) γ-PVDF/Ca-salt (C1), (**d**) α-PVDF/Ca-salt (C2), (**e**) β-PVDF/Ca-salt (C2), and (**f**) γ-PVDF/Ca-salt (C2).

**Figure 10:**
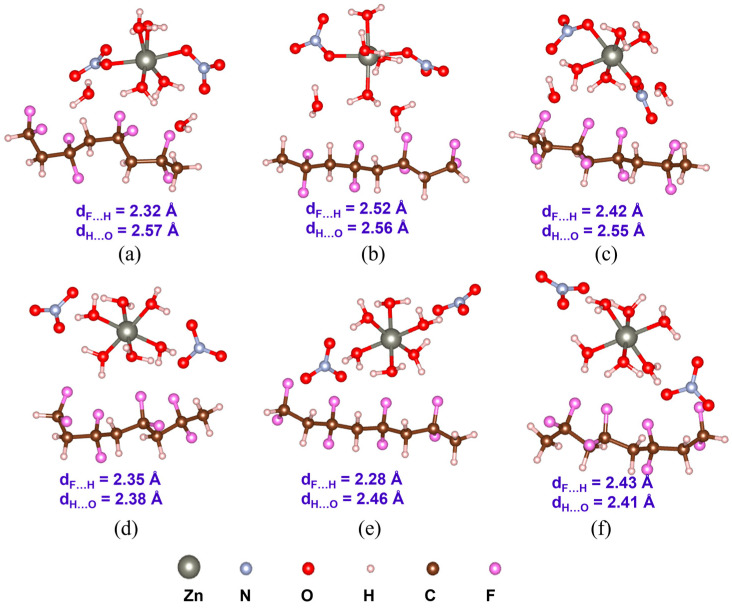
Optimized structures with the average inter-component F…H and H…O distances (d_F…H_ and d_H…O_) of PVDF/Zn(NO_3_)_2_·6H_2_O complexes: (**a**) α-PVDF/Zn-salt (C1), (**b**) β-PVDF/Zn-salt (C1), (**c**) γ-PVDF/Zn-salt (C1), (**d**) α-PVDF/Zn-salt (C2), (**e**) β-PVDF/Zn-salt (C2), and (**f**) γ-PVDF/Zn-salt (C2).

Understanding PVDF/salt interaction is crucial for accurately representing the composite solution of hydrated salts and PVDF. Both C1 and C2 structures of all the salt molecules are considered to elucidate their interaction with α-, β-, and γ-PVDF tetramers. Optimized structures of all the PVDF/salt complexes are provided in Figure 8, Figure 9 and Figure 10. The configurational energy differences according to the salt structures C1 or C2 within the PVDF/salt systems (${∆E}_{PVDF/C}$) and PVDF/salt interaction energies ($E_{int}$) are provided in Table 2. A negative value of ${∆E}_{PVDF/C}$ indicates that the PVDF/C1 complex is more stable than the respective PVDF/C2 configuration. Therefore, PVDF/C1 is more stable than PVDF/C2 for α-PVDF/Ca(NO_3_)_2_·4H_2_O, α-PVDF/Zn(NO_3_)_2_·6H_2_O, β-PVDF/Ca(NO_3_)_2_·4H_2_O, γ-PVDF/Mg(NO_3_)_2_·6H_2_O, and γ-PVDF/Zn(NO_3_)_2_·6H_2_O. Conversely, positive ${∆E}_{PVDF/C}$ indicates that PVDF/C2 is more stable than PVDF/C1 for α-PVDF/Mg(NO_3_)_2_·6H_2_O, β-PVDF/Mg(NO_3_)_2_·6H_2_O, β-PVDF/Zn(NO_3_)_2_·6H_2_O, and γ-PVDF/Ca(NO_3_)_2_·4H_2_O. The configurational stability is further demonstrated by the PVDF/salt interaction energies. A more negative $E_{int}$ indicates stronger inter-component interaction within PVDF/salt complexes. Note that, among all the PVDF/salt systems, β-PVDF/salt systems generally exhibit the strongest PVDF/salt interaction compared to their α-PVDF/salt and γ-PVDF/salt counterparts. This finding aligns with the experimental measurements showing the highest amount of piezoelectric β-PVDF phase in the PVDF/salt samples (Figure 7, Table 1). Specifically, for Ca(NO_3_)_2_·4H_2_O added PVDF samples, the PVDF phase composition was found to be 7.71% α, 92.29% β, and virtually 0% γ. For PVDF/Mg(NO_3_)_3_·6H_2_O samples, the composition was 4.25% α, 95.75% β, and 0% γ. For the PVDF/Zn(NO_3_)_2_·6H_2_O sample, 14.70% α, 85.30% β, and again 0% for γ phases were obtained. These results corroborate an excellent match between the experimental and DFT results.

In order to further investigate the structures of the PVDF/salt systems, the calculated and experimental IR and Raman spectra are compared in Supplementary Materials, Figure S1a–c. Interestingly, substantial consistency is observed between the calculated and experimental IR and Raman spectra (Supplementary Materials, Figure S1a). However, some mismatch between the calculated and experimental results is found at the high-frequency region (beyond 3000 cm^−1^) in the IR spectra of PVDF/salt systems (the raw data are presented in Supplementary Materials, Figure S1b). This discrepancy might be attributed to the solvent effect not being considered in the calculations. Nevertheless, the calculated Raman spectra of PVDF/salt systems show comparatively better alignment with the experimental data (Supplementary Materials, Figure S1c), implying the reliability of the current computational method.

### 3.4. Dielectric Properties

**Figure 11:**
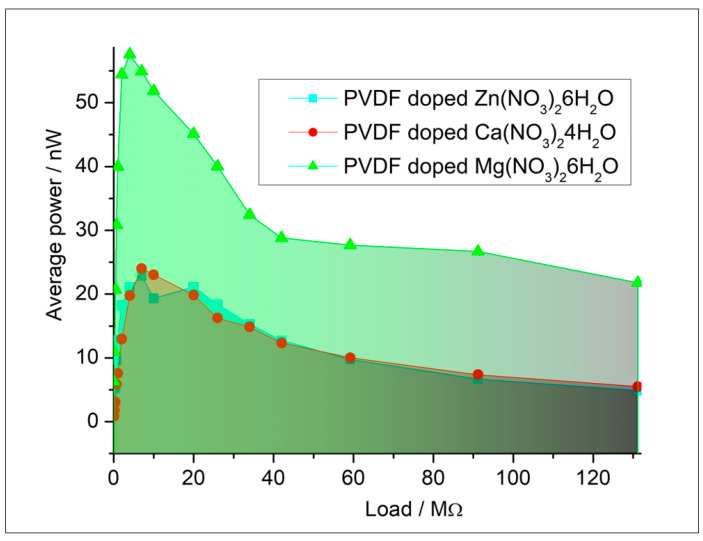
Triboelectric characterization of composites.

The results of triboelectric measurements are plotted to show the relationship of power as a function of the load resistance. Typically, the voltage increases with increasing load resistance until it reaches a saturation point, while the current decreases according to Ohm’s law. The power output curve often reveals a peak, indicating the optimal load resistance for maximum power extraction from the triboelectric generator. The raw data are presented in Supplementary Materials. This analysis helps in understanding the efficiency and performance characteristics of triboelectric materials under varying electrical loads. By systematically varying the load resistance and accurately measuring the corresponding electrical outputs, the triboelectric properties such as maximum power output, optimal load resistance, and overall energy conversion efficiency are thoroughly characterized (Figure 11). The surface roughness of the samples was ~3 µm. The roughness is desirable for triboelectric charge accumulation due to the increased surface area. At the same time, it still provides sufficient contact with the contact for the measurement setup.

**Figure 12:**
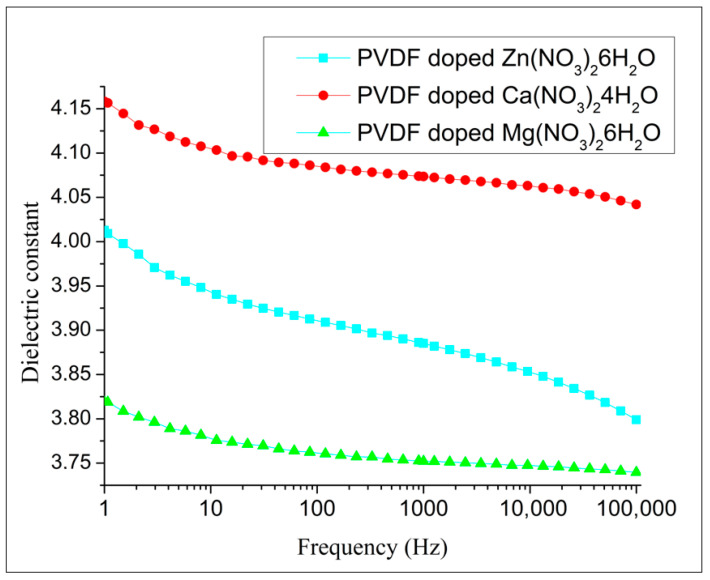
The dielectric constant of composites.

The dielectric characterization of composites reveals that the dielectric constants exhibit highly similar trends (Figure 12). The additional data (dielectric loss, capacity, resistivity) are presented in Supplementary Materials.

Hydrated salts introduce ionic conductivity in the polymer composite, which influences the overall dielectric behavior. Comparing the dielectric properties of PVDF composites with different types of hydrated salts can provide a comprehensive understanding of how different ionic species affect dielectric performance. This can aid in tailoring composites for specific applications. The dielectric constant of PVDF fibers was significantly influenced by the doping of different salt hydrates. Among the tested dopants, PVDF fibers doped with Ca(NO_3_)_2_·4H_2_O exhibited the highest dielectric constant. This increase can be attributed to the effective interaction between the Ca^2+^ ions and the polymer matrix, which enhances the polarization under an electric field. In contrast, PVDF fibers doped with Mg(NO_3_)_2_·6H_2_O showed the lowest dielectric constant. The lower dielectric constant in this case may result from the different ionic size and hydration level of Mg^2+^, which could affect the extent of polarization and the overall dielectric response of the material.

## 4. Discussion

These detailed observations by SEM were crucial for assessing the quality and uniformity of the composite electrospun fibers, providing insights into the optimization of the electrospinning process and the potential applications of the fibers. SEM images (Figure 1a–e) reveal that the fibers exhibit a preferential orientation aligned with the direction of spinning. This alignment indicates that the fibers are consistently arranged parallel to the spinning axis, suggesting uniformity in their structural organization. The detailed images provided by the SEM highlight the extent of this orientation, demonstrating how the spinning process influences the final alignment of the fibers. The SEM images (Figure 1a–e) clearly show this alignment, with fibers consistently arranged in a uniform direction. This uniform alignment is crucial for ensuring consistent electrical properties in the composite fibers. This preferential orientation can impact the properties and overall performance of the fiber material, making it a significant observation in the study of spun fibers [22].

The XRD patterns of the composite samples do not display any diffraction peaks corresponding to Mg(NO_3_)_2_·6H_2_O, Zn(NO_3_)_2_·6H_2_O, and Ca(NO_3_)_2_·4H_2_O. Instead, the diffraction peaks observed are solely attributable to PVDF. This absence of peaks from the nitrate hydrates confirms that the crystal structures of these salts have been disrupted or dissolved within the composite matrix. The PVDF in the composite retains its characteristic phases, which we have comprehensively detailed in our previous work. This observation underscores the effective incorporation of PVDF as the dominant phase within the composite, with the nitrate hydrates no longer maintaining their crystalline integrity.

These different transitions result in four distinct LMM Auger peaks in the spectrum, labeled a, b, c, and d. Each peak corresponds to a specific combination of initial and final states within the L and M shells, resulting in unique energies for the ejected Auger electrons. The presence of Auger peaks for elements reflects the possible electronic transitions between the subshells involved, each contributing to the overall Auger spectrum with its characteristic energy (see Supplementary Materials).

There was an attempt to explain of electrical behavior of the polymeric composites based on the electronegativity of the metal cation in salts. Molecular vibrations observed in FTIR spectra can be linked to the polarizability of the material. Polarizability affects how the material responds to an electric field, thus influencing its dielectric properties. Key types of vibrations include stretching vibrations, which are changes in bond length, and bending vibrations, which are changes in bond angles. Functional groups with polar bonds of PVDF contribute significantly to the dielectric properties due to their dipole moments. Shifts in peak positions and changes in intensity can indicate changes in molecular interactions and polarizability. Two configurations of each nitrate salt are presented. In the initial structures of salt configuration 1, central metal cations (Mg^2+^, Ca^2+^, and Zn^2+^) are coordinated to [NO_3_]^−^ and form [M(NO_3_)_2_], which is surrounded by H_2_O moieties. On the other hand, in the initial structures of salt configuration 2, central metal cations (Mg^2+^, Ca^2+^, and Zn^2+^) are coordinated to H_2_O and form [M(H_2_O)]^2+^ which is surrounded by [NO_3_]^−^ units. However, after optimization, both H_2_O and [NO_3_]^−^ form a bond (ionic or coordination) with the central metal cation. The formation energies of the salts are calculated accordingly. More negative electronic energy and formation energy indicate higher stability of the structure. Notably, Ca(NO_3_)_2_·4H_2_O exhibits high positive formation energy. Interactions between α-, β-, and γ-PVDF and salt configurations are compared. β-PVDF/salt (both configurations 1 and 2) interaction energies are more negative compared to α- and γ-PVDF/salt counterparts. Simulated FTIR and Raman spectra are compared. A mismatch is observed at the high-frequency region which can be caused by hydrogen bond interaction, but it is not valuable for this study.

Independent modeling and experimental characterization by vibrational techniques are in agreement and prove that for obtaining a higher electroactive response, the doping of PVDF fibers by Mg(NO_3_)_2_·6H_2_O is preferable. To confirm this conclusion, the triboelectric measurements were analyzed. Triboelectric charging involves the mechanical transfer of electric charge facilitated by the movement of fiber surfaces. When two solid surfaces make contact and then pull apart, this process results in the transfer of charge from one surface to the other. Having larger polarization of the surface, the PVDF fibers doped by Mg(NO_3_)_2_·6H_2_O showed a higher triboelectric response and could be more desired for practical applications. Comparisons to recent studies are presented in the table and show a wide range of the electro-response (piezo- and tribo-effect) values (Table 3).

The dielectric constant is related to the density and polarizability of polar functional groups identified in the FTIR and Raman spectra. Surprisingly, the dielectric contact of fibers doped by Ca(NO_3_)_2_·4H_2_O shows the highest dielectric constant. The non-polarized α-phase of PVDF can contribute to increased permittivity due to the interplay between its structural characteristics, dipole reorientation, interfacial polarization, and processing conditions. The α-phase has a TGTG’ (trans-gauche-trans-gauche’) conformation, which is non-polar, but still allows the molecular dipoles to reorient under an electric field. This reorientation of dipoles within the PVDF chains increases the polarization of the material, thus contributing to higher permittivity. Although the intrinsic dipole moment is lower due to partial cancellation, the molecular segments can still rotate or reorient under an electric field, enhancing the dielectric response. Additionally, at the interfaces between the crystalline and amorphous regions, charges can accumulate, leading to interfacial polarization, which significantly contributes to the overall permittivity. These effects are further influenced by the processing conditions, such as stretching and poling, which can enhance chain alignment and crystallinity, allowing for better dipole alignment and trapping of charges. The combined effect of these factors results in a significant dielectric response from the α-phase PVDF, even though it is non-polar, leading to an increased permittivity.

## 5. Conclusions

This consistent alignment parallel to the spinning axis signifies a high degree of structural organization, profoundly affecting the mechanical properties and overall performance of the fiber material, which is critical for optimizing the electrospinning process and enhancing the functional applications of the fibers. The successful incorporation of PVDF as the dominant phase, coupled with the dissolution of nitrate hydrates, underscores the efficacy of the composite fabrication method.

Both computational modeling and experimental characterization confirmed that doping PVDF fibers with Mg(NO_3_)_2_·6H_2_O enhances the electroactive response (58 nW), as evidenced by triboelectric measurements indicating superior performance, while fibers doped with Ca(NO_3_)_2_·4H_2_O exhibited the highest dielectric constant (4.04). The non-polarized α-phase of PVDF contributes to increased permittivity through the reorientation of molecular dipoles under an electric field and interfacial polarization at the boundaries between crystalline and amorphous regions.

This complex and comprehensive approach is a way to successfully develop and utilize such composites. The successful utilization and effectiveness of PVDF micro and nanofibers incorporating nitrate salts heavily rely on factors such as the composite’s formulation, structure, and the specific properties of the nitrate salts employed. Achieving optimal results necessitates meticulous design and engineering to customize the material for its intended purpose.

## Figures and Tables

**Table 1:** Configurational energy differences (${∆E}_{C}$), and the formation energies ($E_{f,C1}$ and $E_{f,C2}$) of the salt configurations C1 and C2.

Hydrated Salts	${{∆E}_{C}=E}_{C1}-E_{C2}$ (kcal/mol)	$E_{f,C1}$ (kcal/mol)	$E_{f,C2}$ (kcal/mol)
Mg(NO_3_)_2_·6H_2_O	1.88	−96.20	−312.90
Ca(NO_3_)_2_·4H_2_O	−1.26	−70.10	−315.49
Zn(NO_3_)_2_·6H_2_O	−1.88	−86.88	−313.085

**Table 2:** Configurational energy differences (${∆E}_{PVDF/C}$) of PVDF/salt (C1) and PVDF/salt (C2), and PVDF/salt interaction energies ($E_{int\;[PVDF/C1]}$ and $E_{int\;[PVDF/C2]}$).

PVDF/Salt Complexes	${∆E}_{PVDF/C}=E_{PVDF/C1}-E_{PVDF/C2}$ (kcal/mol)	$E_{int\;[PVDF/C1]}$ (kcal/mol)	$E_{int\;[PVDF/C2]}$ (kcal/mol)
α-PVDF/Mg(NO_3_)_2_·6H_2_O	8.16	−5.54	−14.03
α-PVDF/Ca(NO_3_)_2_·4H_2_O	−0.62	−10.48	−11.05
α-PVDF/Zn(NO_3_)_2_·6H_2_O	−1.26	−9.11	−12.59
β-PVDF/Mg(NO_3_)_2_·6H_2_O	9.41	−10.59	−18.42
β-PVDF/Ca(NO_3_)_2_·4H_2_O	−2.51	−21.55	−20.31
β-PVDF/Zn(NO_3_)_2_·6H_2_O	7.53	−9.20	−18.66
γ-PVDF/Mg(NO_3_)_2_·6H_2_O	−2.51	−15.85	−11.56
γ-PVDF/Ca(NO_3_)_2_·4H_2_O	0.62	−10.46	−12.48
γ-PVDF/Zn(NO_3_)_2_·6H_2_O	−2.51	−17.22	−16.85

**Table 3:** Ranking of electro-response in recent publications.

Material	Pressure	Voltage	Current	Reference
PVDF + (Zn(NO_3_)_2_·6H_2_O)	22.7 kPa	0.4 V	57 nA	This study
PVDF + (Ca(NO_3_)_2_·4H_2_O)	22.7 kPa	0.41 V	58 nA	This study
PVDF + (Mg(NO_3_)_2_·6H_2_O)	22.7 kPa	0.48 V	120 nA	This study
PVDF + ZnO	∼1.13 kPa	0.51 ± 0.12 V	∼190 nA	[23]
PVDF + MXene	200 kPa	3.15 V	134 nA	[24]
PVDF + NiO	9.81 Pa	5.5 V	1830 nA	[25]
PVDF + CsPbBr_3_	100 MPa	120 V	35 µA	[26]
PVDF + Cu@AgNP	7.3 MPa	0.147 V	0.42 nA	[27]
PVDF + cellulose nanocrystal	15 kPa	0.146 mV	0.009 nA	[28]

## Data Availability

The data are available and submitted with this article.

## Supplementary Materials

The following supporting information can be downloaded at: https://www.mdpi.com/article/10.3390/polym16172412/s1, Figure S1a—Experimental (red) and simulated (black) vibrational spectra of pristine PVDF; Figure S1b—Experimental (red) and simulated IR spectra (black) of PVDF/salt systems; Figure S1c—Experimental (red) and simulated (black) Raman spectra of PVDF/salt systems.
