# Supplementary material for: Correlation of Dielectric Properties and Vibrational Spectra of Composite PVDF/Salt Fibers
Source: Polymers (Basel). 2024 Aug 26;16(17):2412. doi: 10.3390/polym16172412 (PMC11396973; doi:10.3390/polym16172412)
Supplement: Supplementary file 1 [file polymers-16-02412-s001.zip › polymers-3129085-supplementary.pdf]

Correlation of Dielectric Properties and Vibrational Spectra of Composite PVDF/Salt Fibers

Rashid Dallaev [1,*], Ranjini Sarkar [2], Daud Selimov [3], Nikola Papež [1], Pavla Kočková [1], Richard Schubert [1], Klara Částková [4], Farid Orudzhev [3], Shikhgasan Ramazanov [5] and Vladimír Holcman [1]

[1] Department of Physics, Faculty of Electrical Engineering and Communication, Brno University of Technology, Technická 2848/8, 616 00 Brno, Czech Republic; nikola.papez@vut.cz (N.P.); xsneubauerovap@vut.cz (P.K.); xschub01@vut.cz (R.S.); holcman@vut.cz (V.H.)

[2] Department of Metallurgical and Materials Engineering, Indian Institute of Technology Kharagpur, Kharagpur 721302, West Bengal, India; ranjinisarkar.kgp.1991@gmail.com

[3] Department of Inorganic Chemistry and Chemical Ecology, Dagestan State University, St. M. Gadjieva 43-a, 367015 Makhachkala, Russia; farid-stkha@mail.ru (F.O.); daud-selimov@live.com (D.S.)

[4] Central European Institute of Technology BUT, Purkyňova 123, 612 00 Brno, Czech Republic; klara.castkova@ceitec.vutbr.cz

[5] Amirkhanov Institute of Physics, Dagestan Federal Research Center, Russian Academy of Sciences, 367003 Makhachkala, Russia; ramazanv@mail.ru

* Correspondence: rashid.dallaev@vut.cz

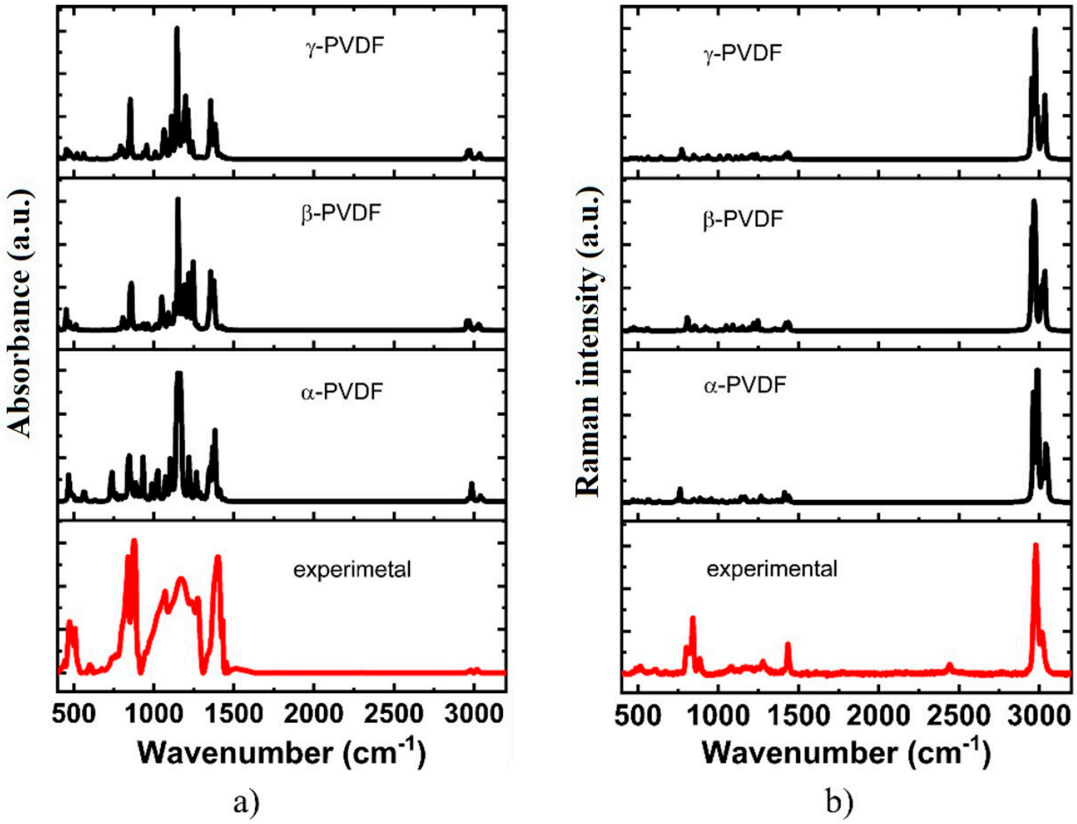

Figure S1a. Experimental (red) and simulated (black) vibrational spectra of pristine PVDF: a) IR spectra and b) Raman spectra.

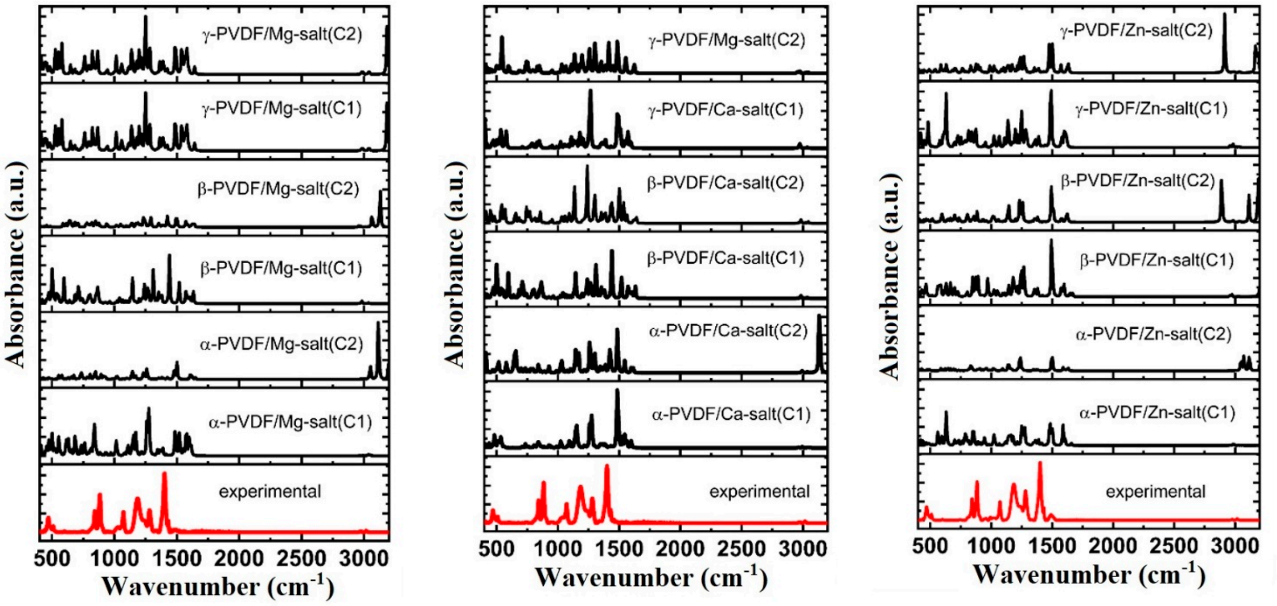

a) b) c)

Figure S1b. Experimental (red) and simulated IR spectra (black) of PVDF/salt systems: a) PVDF/$Mg(NO_3)_2 \cdot 6H_2O$, b) PVDF/$Ca(NO_3)_2 \cdot 4H_2O$, and c) PVDF/$Zn(NO3_3)_2 \cdot 6H_2O$).

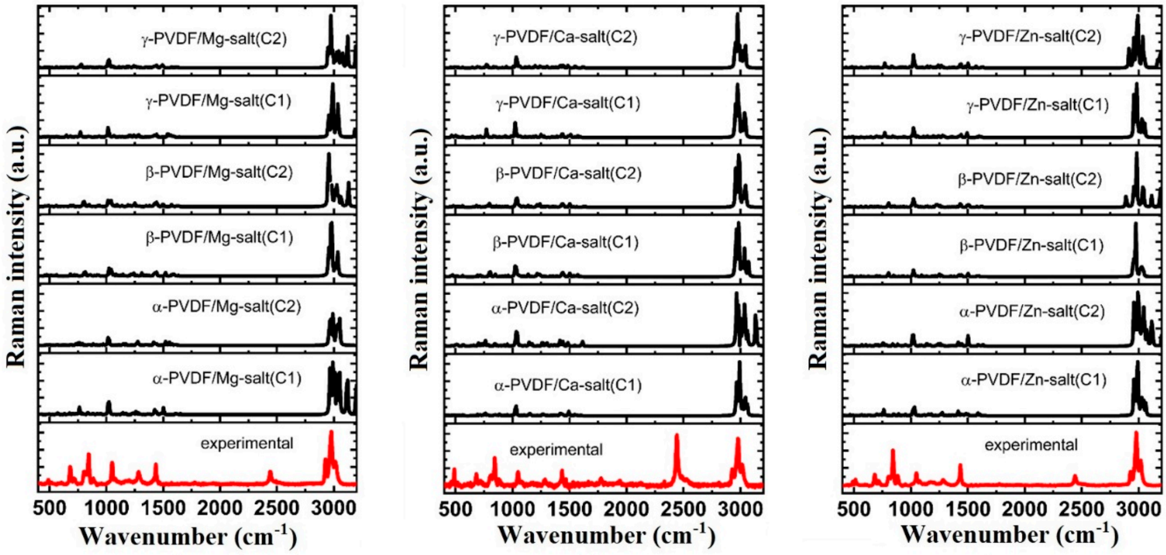

a) b) c)

Figure S1c. Experimental (red) and simulated (black) Raman spectra of PVDF/salt systems: a) PVDF/$Mg(NO_3)_2 \cdot 6H_2O$, b) PVDF/$Ca(NO_3)_2 \cdot 6H_2O$, and c) PVDF/$Zn(NO_3)_2 \cdot 6H_2O$.
